# Psychometric inspection of an internalized homonegativity measure

**DOI:** 10.3389/fpsyg.2025.1569382

**Published:** 2025-09-09

**Authors:** Matthew James Kerry, Andreas Pfister, Paula Krüger

**Affiliations:** ^1^Department of Health, Zurich University of Applied Sciences, Winterthur, Switzerland; ^2^Department of Social Work, Lucerne University of Applied Sciences and Arts, Lucerne, Switzerland

**Keywords:** internalized homonegativity, sexual identity, LGBTQ persons, structural validity, cross-linguistic validity, discriminant validity

## Abstract

Internalized homonegativity (IH) is a substantively important construct linked to various health-related quality-of-life indicators. Despite IH’s prominence in the homosexuality literature, however, several measurement challenges are posed to advancing its empirical evidence base. This study aimed to bring modern psychometric methods to bear on a measure of IH in a general population sample of self-reported gay, lesbian, and bisexual respondents in Switzerland (*n* = 988). Specifically, we used a prospective observational cross-sectional design with questionnaire methodology to examine three aspects of the validity of a 7-item IH instrument: (1) structure validity, (2) cross-linguistic validity, and (3) “known-groups” (discriminant) validity. Our findings indicated support for the 7-item IH measure’s essential unidimensionality. Furthermore, we found support for IH’s measurement equivalence across German- and French-speaking regions, whereas mixed support was found for its extension to bisexual respondents. Finally, the IH measure exhibited discriminant validity, such that depression and poor self-reported health status were associated with higher IH scores. In conclusion, the IH instrument may be used as a unidimensional measure across German- and French-speaking general populations; however, further research should focus on extending its linguistic validity and measurement equivalence to bisexual and transgender populations.

## 1 Introduction

Meyer and Dean (1998) defined internalized homophobia as “the gay person’s direction of negative social attitudes toward the self, leading to a devaluation of the self and resultant internal conflicts and poor self-regard” (p. 161). However, the term “internalized homophobia” has been critiqued and further differentiated. Wider societal factors shape the devaluation of the self. Internalized homophobia “…is not simply a product of personal, subjective, and ‘irrational’ fears” (Berg et al., 2016, p. 542). Thus, the term “internalized homonegativity” (IH) may be more appropriate and is increasingly used in contemporary scientific literature (see, e.g., Badenes-Ribera et al., 2018; Lefevor et al., 2023).

In Europe, the situation for LGBT+ persons varies greatly across sovereign states. The importance of IH for oppression, stigmatization, and minority stress is relevant to Switzerland as a whole (Krüger et al., 2023). For example, minority stress theory has postulated that marginalized intersections (racial gay minorities, or immigrant gay minorities) may be particularly at-risk of elevated IH (Frost and Meyer, 2023). Pertinent to the current study, minority stress theory posits that stigmatized individuals suffer negative health outcomes as a result of enduring societal discrimination-borne chronic stress. Health disparities between marginalized group members include the domains of physical health, substance abuse, and mental health.

Empirical research has shown IH to be associated with depression (Davidson et al., 2017), anxiety (DiPlacido, 1998), and substance use disorders (Moody et al., 2018). IH has also exhibited meta-analytic evidence for its association with bodily preconceptions (Badenes-Ribera et al., 2018) and suicidal ideation (Williams et al., 2023). IH has also been found to be related to aggression (Berg et al., 2016), partner violence (Badenes-Ribera et al., 2019), sexually transmitted infections (Berg et al., 2015), and intergroup relations (van Leeuwen et al., 2016). The negative consequences of IH are postulated to result from a psychological dilemma, specifically, a self-image disjuncture between romantic desires and public shame-borne feelings of belittlement and low self-esteem (Berg et al., 2016).

Despite IH’s substantive correlations with various health-related quality-of-life indicators, challenges remain regarding measurement consistency. For example, a systematic mapping review of empirical research identified nine distinct instruments (Berg et al., 2016). Although past studies have purported to measure IH’s measurement using 1-factor, 2-factor, or 3-factor structures, recent modern psychometric evidence has provided evidence for a 1-factor (unidimensional) solution (Wickham et al., 2021). It should also be noted that the differing number of factor solutions may simply be owed to sample variation rather than instrument instability. Indeed, given our diverse target sample of Switzerland—with several official national languages and cultures—we aimed to validate a simpler instrument that may be more robust to sampling variation, rather than an overly specific instrument that may be less generalizable. A unidimensional solution may be advantageous for both administrative and analytical ease, as well as for minimizing response burden via shorter instruments and fewer items (Reeve, 2003).

Given previous inconsistency regarding IH’s latent-factor structure, the current study aims to bring modern psychometric evidence to bear on a previously validated, adapted 7-item IH measure for use in Switzerland (Smolenski et al., 2010). To balance construct coverage (breadth) and brevity, the first two items from each of Smolenski’s three factors of IH were adopted. A seventh item from the original moral subfactor was added based on the author’s subject matter expert knowledge of the potential applicability within Switzerland. Specifically, the current study examines the following four hypotheses pertaining to IH’s internal structure validity, cross-linguistic validity, and “known-groups” (sensitivity) validity.

*H1:* We hypothesize that the IH measure will exhibit essential unidimensionality.

*H2:* We hypothesize that the IH measure will exhibit sufficient cross-linguistic validity evidence, defined as the overall measure indicating small practical effect sizes (Cohen’s *d* < 0.20) across German and French languages in Switzerland.

Empirical mapping of IH has exhibited positive associations with depression (Berg et al., 2016). Additionally, a recent meta-analysis of *k* = 68 studies, yielding *n* = 151 effect sizes, found that IH is negatively associated (*r* = −0.28) with general health status (Lefevor et al., 2023). Based on these findings, we hypothesized the following “known-groups” validity test for our locally developed IH instrument:

*H3:* We hypothesize that the IH measure would be positively associated with depression scores.

*H4:* We hypothesize that the IH measure would be negatively associated with general health status.

We examined H3 and H4 using both median-split t-tests to compare high- and low-IH score groups (i.e., “known groups” discriminant validity), as well as correlational analysis.

## 2 Methods

### 2.1 Sample

In total, 2,064 lesbian, gay, bisexual, and transgender (including non-binary) individuals participated in the study. The survey was available in German, French, Italian, or English. Although “Romansch” is the national language of Switzerland, it is spoken by only 0.4% of the population, and most Romansch speakers are also fluent in German. Due to minimal model-identification requirements (*n* > 100), only the German and French language versions of the IH measure were included in the current psychometric examination. In total, *n* = 1,174 eligible respondents self-identified as German- or French-speaking and as gay, lesbian, or bisexual. Twenty-three respondents (*n* = 23) were missing data on the focal IH measure in the system and were listwise deleted from the dataset. Due to space limitations, the full missing data analysis and data cleaning technical report is listed in Supplementary material. An additional 163 respondents were identified as careless based on extreme responding across normal- and reverse-scored items and were removed, thereby reducing the effective sample to *n* = 988 for analyses.

### 2.2 Design

Responses were collected via purposive sampling in a Swiss-national cross-sectional survey. Specifically, potential participants were recruited via advertisements in traditional and social media outlets by the universities, the Federal Office of Public Health, and LGBT organizations (e.g., TGNS, LOS, and Les Klamydia’s, Pink Cross). In addition, sampling frames included selected institutions from the healthcare sector, which were asked whether they could draw attention to the study on their homepage and/or by flyer display. A cross-sectional design with survey methodology was conducted (see Krüger et al., 2023, for details). On behalf of the Federal Office of Public Health, data were collected from mid-May to mid-July 2021 in a national survey aimed at describing the health status and healthcare access of LGBT people living in Switzerland.

### 2.3 Measures

Several socio-demographic data were collected for the present study, including sexual identity, defined as the person’s self-identification (e.g., gay, lesbian, and bisexual) (Patterson et al., 2017). The focal measure of the current short report is a 7-item Internalized Homonegativity Scale. It is adapted from Smolenski et al.’s (2010) 7-item revised Reactions to Homosexuality scale. Specifically, 6 of the 7 items were adopted and underwent standard forward-back translation protocols with native speakers of German and French. The final item from the original first subscale was replaced with one of Smolenski’s items from his fourth factor to increase construct breadth and content-relevance, based on the authors’ knowledge of the Swiss population, “Homosexuality is morally acceptable to me.” Respondents indicate their agreement on a 6-point Likert-type scale ranging from “strongly disagree” (1) to “strongly agree” (6). An example item is “I feel comfortable in gay/lesbian bars.” Internal consistency, indexed by McDonald’s omega (*ω*) reliability coefficient, was adequate at *ω* = 0.85. In addition, we administered a single-item self-reported health status measure on a 6-point Likert-type scale (very poor – very good). We also administered the 9-item Patient Health Questionnaire for assessing depression, which also exhibited strong internal consistency reliability in the current study, *ω* = 0.88 (Kroenke et al., 2001). Responses to the Patient Health Questionnaire are recorded on a 4-point Likert-type scale ranging from “not at all” (1) to “nearly every day” (4).

### 2.4 Analyses

Data cleaning and classical analyses were conducted in software IBM SPSS v29 (IBM Corp., 2023). Specifically, missing data, parallel analyses, and maximum likelihood estimation were conducted (O’Connor, 2000). Unidimensional indices were computed using Dueber’s (2017) bifactor indices calculator. Cross-linguistic validity was assessed using modern psychometric analyses conducted in software IRTPRO v5.1 with graded response model specifications (Cai et al., 2020). Specifically, differential item functioning (DIF) or, item bias, testing proceeded with magnitude assessment, comprising conventional effect size (ES) estimation according to Cohen’s (1988) rubric of small (*d* ≥ 0.20), medium (*d* ≥ 0.50), and large (*d* ≥ 0.80) using VisualDF (Meade, 2010). “Known-groups” validity was tested using an independent t-test on median splits of the grouping variables self-rated health and depression.

## 3 Results

Summary sample descriptive characteristics are presented in Table 1. Univariate item-level descriptive statistics, frequency response patterns, and graphical inspection of scale-level normal Q–Q plots provided tentative evidence for inferring univariate-normal distributional assumptions.

**Table 1:** Summary of descriptive characteristics.

Characteristic	*M* (*SD*), *n* (%)
Age (years)	37.74 (13.69)
Birth sex	
Female	419 (42%)
Male	569 (58%)
Sexual identity	
Lesbian	409 (41%)
Gay	311 (32%)
Bisexual	268 (27%)
Language	
German	709 (72%)
French	279 (26%)
Internalized homonegativity	5.24 (0.67)

*N = 988.*

Specifically, nearly all items’ skewness (<3) and kurtosis (<7) values were within normality-range recommendations for large sample sizes (*n* > 300) (see Table 2). Item 6, however, exhibited non-normal descriptive and may be a candidate for exclusion in future studies. This item was retained in the current study. Therefore, analyses proceeded with parametric tests.

**Table 2:** Descriptive statistics and factor loading of the IH items.

	*M* (*SD*)	*Mdn*	Skew	Kurtosis	Floor %, Ceiling %	Factor loadings
Item 1_1_	4.62 (1.25)	5	−0.90	0.47	4%, 10%	0.38
Item 2_1_	5.26 (1.41)	6	−2.08	3.21	55%, 9%	0.19
Item 3_2_	5.25 (1.26)	6	−1.98	3.42	5%, 54%	0.47
Item 4_2_	5.10 (1.15)	5	−1.47	20.07	3%, 42%	0.63
Item 5_3_	5.21 (1.04)	6	−1.59	2.75	1%, 38%	0.77
Item 6_3_	5.86 (0.54)	6	−5.90	43.45	1%, 88%	0.51
Item 7_3_	5.41 (1.12)	6	−2.28	50.00	3%, 62%	0.61
Total-average	5.24 (0.67)	5.33	−1.70	5.28	2%, 0.1%	

*N* = 988, maximum likelihood estimation for factor analysis. Low Italian (*n* = 65) and English respondents (*n* = 57) also exhibited normally distributed data assumptions on the IH measure. (skew < 3, kurtosis < 7). Subscripts indicated factor loadings in the comparative CFA test for 2- and 3-factor models. Only unidimensional (1-factor) loadings are displayed herein.

### 3.1 Structural validity

**Figure 1:**
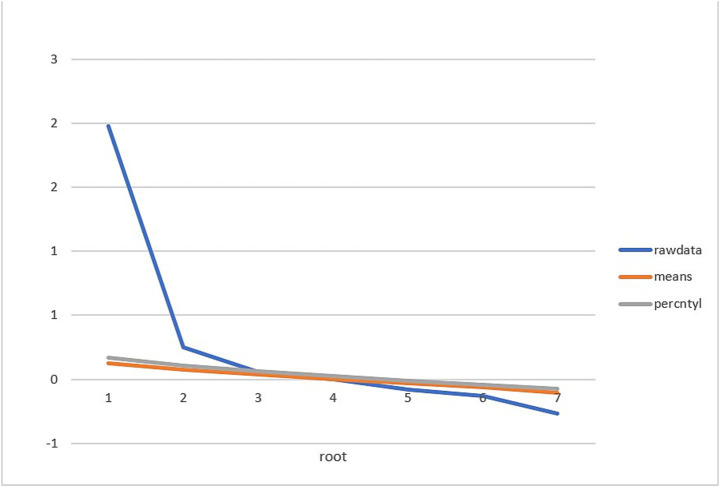
Parallel analysis for latent-factor structure. Raw data, raw-eigenvalue estimates; means, mean-eigenvalue estimates; percentile, 95% confidence interval eigenvalue estimates.

Parallel analysis of raw data suggested a potential 2-factor solution for extraction (Figure 1). This was, however, further scrutinized with exploratory factor analysis using maximum likelihood estimation, which extracted only a single factor. Specifically, the first/s eigenvalue ratio was computed as 2.67/0.97 = 2.75, suggesting negligible multidimensionality in the total sample. Finally, a bifactor model was estimated in order to compute hierarchical-omega (ωH) values for the general factor, which indicates the percentage (%) of reliable variance that can be contributed to the general factor. The current IH scale exhibited ωH = 0.90, supporting the 7-item scale’s interpretation as “essentially unidimensional” (ωH > 0.80; Reise et al., 2013, p. 224). Given past-reported factor solutions, however, we also used a confirmatory factor analysis to compare our 1-factor to 2- and 3-factor solutions as well. Items loaded onto previously reported “source” factor-loading solutions (Smolenski et al., 2010). A 1-factor model exhibited good fit to the data, *χ*^2^(488) = 1,603.53, *p* < 0.001, RMSEA = 0.05, BIC = 16,344.61. A 2-factor model exhibited an adequate, but comparatively worse fit to the data, *χ*^2^(487) = 1,602.71, *p* = 0.371, RMSEA = 0.06, BIC = 16,480.12. Finally, a 3-factor model also exhibited an adequate, but comparatively worse fit to the data, *χ*^2^(486) = 1601.19, *p* = 0.224, RMSEA = 0.06, BIC = 16,494.29. These findings support the retention of our 1-factor measures of IH.

### 3.2 Cross-linguistic validity

The two-step procedure for item bias (differential item functioning, DIF) proceeded with statistical detection. Unsurprisingly, given the relatively large sample size (*n* = 988), traditional statistical indices detected nominally significant DIF across five out of seven items, although only two items indicated DIF on the slope parameter (see Table 3). Step 2’s magnitude assessment proceeded with computation of ES standardized differences (ESSDs). As exhibited in Table 3, large ESs were observed for reversed-coded item 2 and item 3. All other ESs were small according to Cohen’s (1988) rubric. Indeed, the IH-overall score indicated an ESSD of only 0.11. These findings support IH cross-linguistic validity across Swiss-German-speaking and Swiss-French-speaking samples.

**Table 3:** Summary DIF statistics by total and slope parameter estimates for language.

Statistical DIF							Practical DIF
Item	*X^2^*_(total)_	*df*	*p*-value	*X^2^*_(slope)_	*df*	*p*-value	ES Standardized Difference (ESSD)
Item 1	17.0	6	0.012	0.2	1	0.631	0.361
Item 2_R_	66.4	6	0.000	40.0	1	0.052	1.801
Item 3	70.4	6	0.000	14.6	1	0.000	−0.774
Item 4	26.4	6	0.000	3.2	1	0.074	−0.073
Item 5	7.5	6	0.281	1.6	1	0.203	0.122
Item 6	4.1	5	0.543	0.2	1	0.684	0.024
Item 7	13.4	6	0.041	9.4	1	0.001	−0.171
							*Overall Score ESSD* = 0.112

*N = 988. Anchored on all items. R = reverse-scored.*

**Figure 2:**
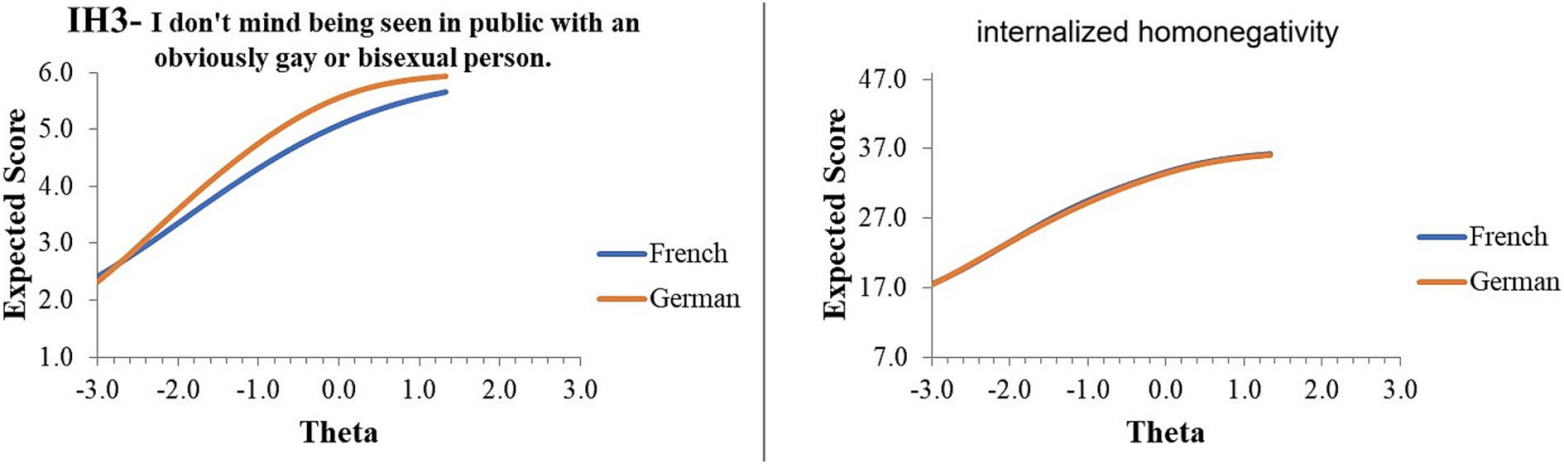
Item and instrument response curves.

Excluding the likely reverse-scored artifact item, the highest practical DIF and overall DIF of the IH measure are illustrated in Figure 2. As shown, on average, French respondents with equal estimated standing as German respondents on latent-IH scored approximately 0.40 points lower in agreement with the item, “I don’t mind being seen in public with an obviously gay/lesbian or bisexual person.” The overall measure, however, indicated very little difference between German- and French-language versions. Taken together, little evidence for meaningful item bias was detected in the current sample, although future research should continue to examine extensions into additional languages.

### 3.3 Known-groups validity

**Figure 3:**
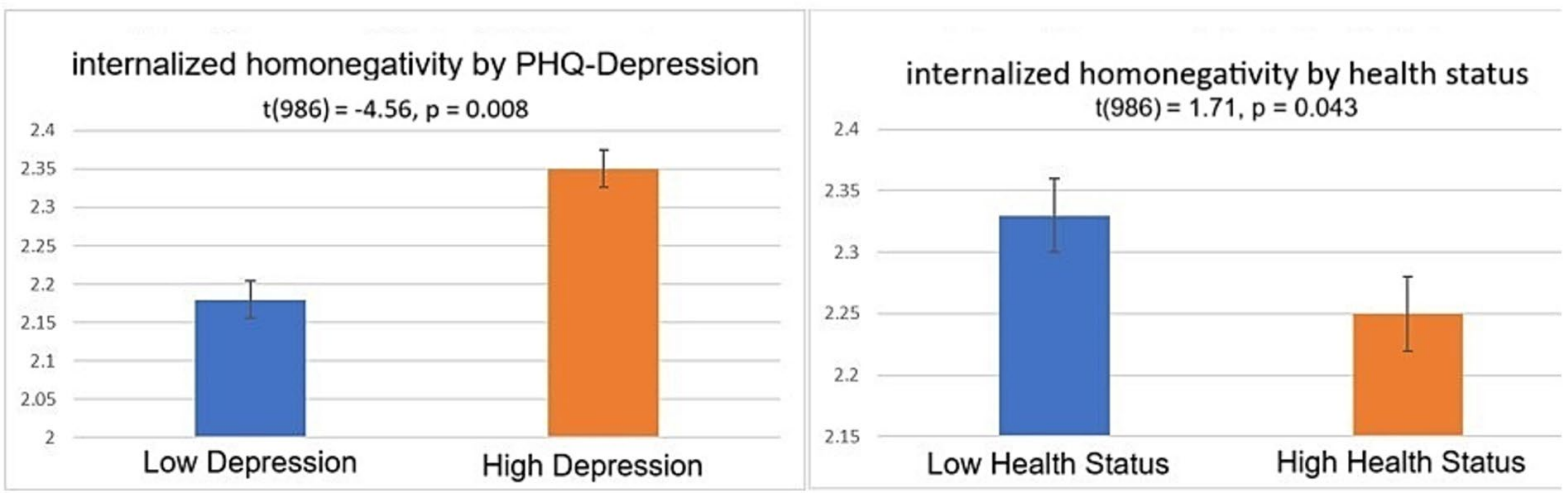
Independent t-tests of internalized homonegativity by **(A)** depression and **(B)** health status. “Low” and “High” groups correspond to median splits on the focal variables. *N* = 988.

Independent-sample t-tests were conducted based on median splits of focal variables, namely PHQ-Depression and self-reported health status. The t-test for depression was significant in the hypothesized direction, *t*(986) = −4.56, *p* = 0.008, with an additional effect size calculated without group classification, *r* = 0.14, *p* < 0.001. The t-test for health status was also significant in the hypothesized direction, *t*(986) = 1.71, *p* = 0.043, with an additional effect size calculated without group classification*, r* = −0.10, *p* = 0.002. These findings support hypotheses 3 and 4 for the IH measure’s sensitivity across known groups. The T-test results are illustrated in Figure 3.

### 3.4 Exploratory analyses

Because we extended the current IH measure to bisexual populations by adapting item wording, we explored whether measurement equivalence holds in this new group. Although this was not the focus of the current paper, we repeated the two-step DIF assessment above to detect potential item bias resulting from adapting the wording for the IH measurement among bisexual respondents. The summarized results, reported in Table 4, indicated that more items were detected for potential DIF (4 of 7). Step 2’s magnitude assessment indicated that three items exhibited medium to large DIF, whereas item 5 exhibited small DIF.

**Table 4:** Summary DIF statistics by total and slope parameter estimates for homosexual vs. bisexual.

Statistical DIF							Practical DIF
Item	*X^2^*_(total)_	*df*	*p*-value	*X^2^*_(slope)_	*df*	*p*-value	ES Standardized Difference (ESSD)
Item 1	32.2	6	0.000	31.1	1	0.000	0.671
Item 2_R_	58.6	6	0.000	57.2	1	0.000	−3.821
Item 3	21.8	6	0.000	19.3	1	0.000	0.761
Item 4	10.0	6	0.993	0.9	1	0.971	0.005
Item 5	21.5	6	0.000	19.2	1	0.000	−0.366
Item 6	4.5	5	0.482	1.5	1	0.822	0.058
Item 7	10.0	6	0.983	1	1	0.964	0.048
							*Overall Score ESSD* = 0.070

*N = 988. Anchored on all items. R = reverse-scored.*

These findings need further examination in a larger sample.

**Figure 4:**
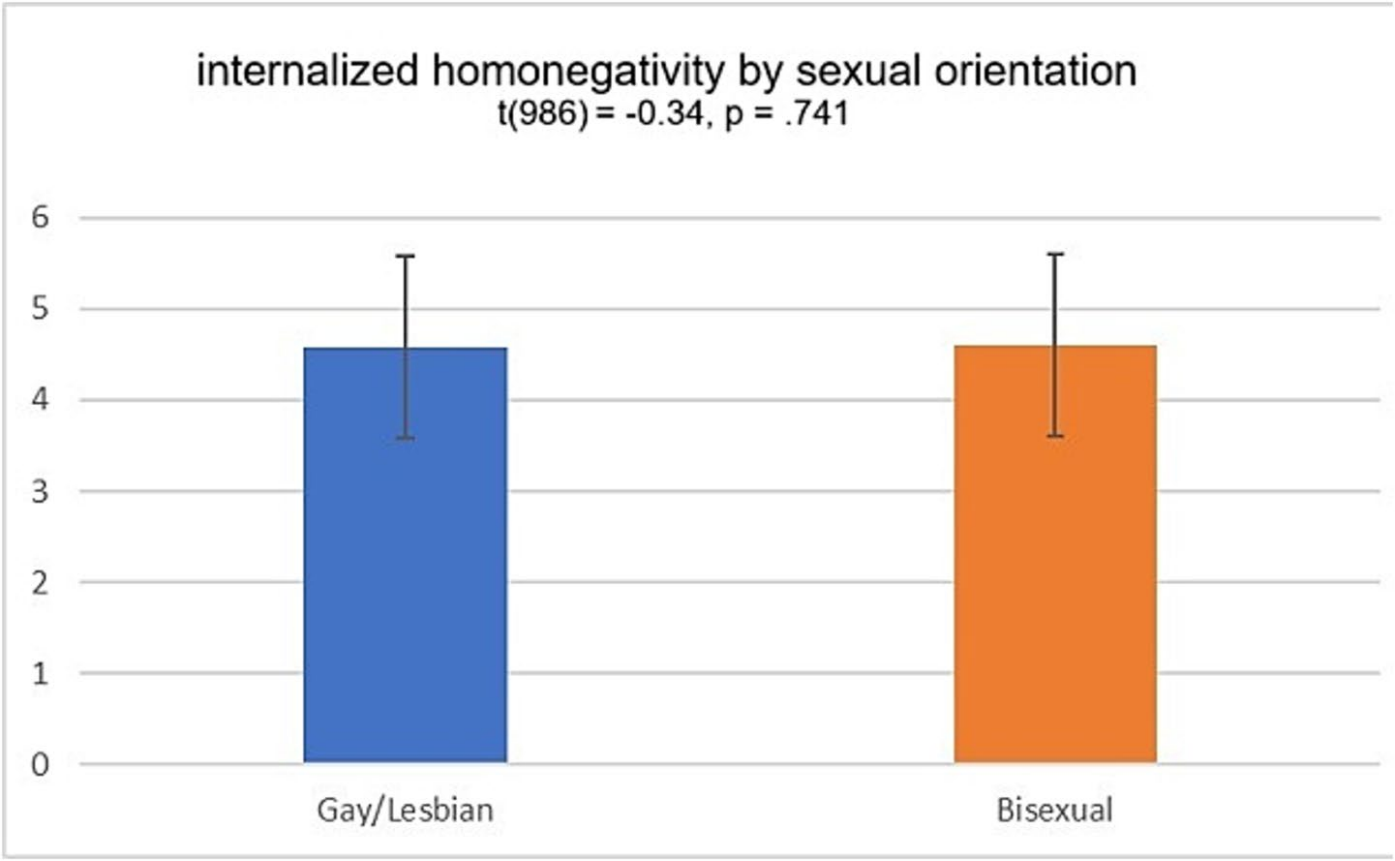
Independent t-test of internalized homonegativity by sexual orientation. *N* = 988.

Finally, an independent *t*-test indicated no significant difference between sexual identity, *t*(986) = −0.34, *p* = 0.741 (Figure 4).

## 4 Discussion

This study aimed to bring psychometric evidence to bear on a measure of internal homonegativity. Specifically, four hypotheses were tested as follows: (1) IH would exhibit essential unidimensionality, (2) IH would exhibit cross-linguistic validity, (3) IH would positively associate with depression, and (4) IH would negatively associate with health status. Test results of the hypotheses are summarized below.

First, regarding dimensionality, our parallel analysis results indicated that the IH exhibited a strong general factor, which was confirmed by follow-up exploratory factor analysis. Furthermore, bifactor indices were calculated to compute a hierarchical-omega (ωH) coefficient for the general factor, which satisfied the condition for “essential unidimensionality.” Therefore, IH scores are interpretable as unidimensional construct indicators in future research. This finding is consistent with recent modern psychometric results (Wickham et al., 2021).

Second, regarding cross-linguistic validity, we conducted a statistical and practical item bias (DIF) assessment. Statistical findings indicated two potentially DIF items of the IH. Follow-up practical assessment revealed only one of these items to be potentially problematic, with an effect size *d* > 0.50. The follow-up assessment further revealed, however, that the reverse-scored item exhibited a large DIF, although we recommend retention of this item for potential “careless responding” detection in future research (Ward and Meade, 2023). The overall IH exhibited minimal practical DIF, suggesting sufficient cross-linguistic validity across Swiss-German and Swiss-French administrations.

Third, independent t-tests were conducted to test “known-groups” differences on the IH measure. Consistent with the literature linking IH to depression, IH scores were significantly higher for high-depression groups compared to low-depression groups (Newcomb and Mustanski, 2010). Fourth, and complementary to this, in accordance with the literature supporting the health benefits of low IH, IH scores were significantly lower among individuals reporting better health status (Lefevor et al., 2023).

Practically, the IH measure may be administered in both German- and French-speaking parts of Switzerland, whether in clinical or general population settings. Its brevity (7-item) offers benefits to aggregate public health monitoring and research endeavors. Further validation in clinical settings should be conducted and is elaborated in detail below.

## 5 Limitations and future research

Cross-linguistic validity can and should be extended to additional language regions (Italian and English). Although our sample sizes were low for Swiss-Italian (*n* = 65) and English-speaking (*n* = 57) respondents in Switzerland, the preliminary findings reported in Table 3 provide tentative evidence for the appropriateness of administering the IH measure in more language regions. These tentative findings should be further examined using larger and more representative samples. Furthermore, potential “self-presentation” of the homonegativity self-report may be examined for convergent validity with validated implicit measures, such as conditional reasoning tests (James and LeBreton, 2010) or through other-reported measures for corroboration. For example, respondents may not acknowledge experiencing IH (Lord, 1980), yet they still exhibit negative consequences associated with latent IH. The self-report may be triangulated to understand the construct representation in IH questionnaires. Furthermore, future research should focus on extending the validity evidence of the IH measure to additional domains, such as responsiveness over time and criterion validity. The reverse-scored item may be a crude way of identifying careless responders, and this should be more deeply examined by response pattern indices. Relatedly, item 6 “Homosexuality is morally acceptable to me” exhibited negative skew and high leptokurtosis. Given that 97% of respondents replied in the highest two response categories, this item may reflect outdated perceptions and may be a candidate for deletion in future studies. The shortening of the instrument in the current sample indicated minimal compromise to internal consistency reliability with a 6-item estimate of *ω* = 0.78. Finally, it should be noted that linguistic equivalence does not necessarily equate to cultural equivalence. Although our DIF analyses indicated linguistic equivalence, further content validation studies (cognitive interviews, pilot tests) should be conducted in future studies.

## 6 Conclusion

The IH measure demonstrated essential unidimensionality, sufficient cross-linguistic validity between German and French language versions, and “known groups” validity. Further linguistic validations may be prioritized, but preliminary evidence supports the IH measure as a short, reliable, and tentatively valid instrument for future public health research.

## Data Availability

The data was provided by the Federal Office of Public Health and is available to researchers upon written request.
