# Supplementary material for: Psychometric inspection of an internalized homonegativity measure
Source: Front Psychol. 2025 Sep 9;16:1569382. doi: 10.3389/fpsyg.2025.1569382 (PMC12456324; doi:10.3389/fpsyg.2025.1569382)
Supplement: Supplementary file 1 [file Supplementary_file_1.pdf]

Supplementary Material

1 LGBT$^+$ Missing Data & Cleaning Analysis – Technical Short Report

A total of *n* = 1,192 responses were recorded. *N* = 11 did not give consent and were automatically removed from the dataset (*n* = 1,181). Seven participants identified as missing all datum (100% incomplete, i.e., system-missing, or unit nonresponse) were removed from the dataset (*n* = 1,174). A further twenty-three participants only completed sociodemographic variables but were system-missing on all IH items and, so, were removed from the dataset (*n* = 1,151). Of the remaining *n* = 1,151 participants, a series of Little's (1988) missing-completely-at-random (MCAR) chi-square tests was conducted on IH items to provide statistical inference regarding the nature of the expected change vis-à-vis potential nonresponse bias. Given the relatively large sample size (n), we proceeded with interpretation of p-values at < 0.01 significance levels.

Table A1. Little's MCAR test results.

IH Item	% Missing	Little MCAR (DF), p-val	Imputed Values
Item 1	7.5%	.449(2), *p* = 0.799	4.62 / 5
Item 2	.2%	n/a, median-impute	6
Item 3	.2%	n/a, median-impute	6
Item 4	.4%	n/a, median-impute	5
Item 5	.5%	n/a, median-impute	6
Item 6	.5%	n/a, median-impute	6
Item 7	3.1%	10.36(2) = 0.01	5.41 / 5

Based on above-tabled results, imputed values are implemented. Given the negligible amount of missing data for IH items 2 – 6 (< 1%), listwise deletion or median-imputation is advisable and, so, median imputation is currently implemented.

For items Item 1 and Item 7, Little's MCAR's were conducted with the adjacent two items in order to balance the missing-data pattern across the instrument. For example, Item 1 was tested with items 2 and 3, and Item 7 was tested with items 5 and 6. Results indicated non-significant MCARs, suggesting that the missing data mechanism is completely random and so imputation for these two items proceeded with the expectation-maximization method.

1.1 Careless Responders

We computed a careless response index based on extreme-category responding across the first three IH items. Given that Item 2 is reverse scored, this three-item response pattern index gave us an initial starting place to identify potential careless responders. This was cross-checked with "time on survey", which indicated that all flagged respondents were in the upper 5% of fastest completion times of the survey, supporting careless responder inferences. Taken together, $n = 163$ participants were identified as careless responders based responding consistently in extreme categories to Item 1, Item 2(R), and Item 3 and were removed from the dataset ($n = 988$).

The median time to complete the survey was approximately twenty-seven minutes.

1.2 References

Bennett, D. A. (2001). How can I deal with missing data in my study? Australian and New Zealand journal of public health 25 (5), 464–469.

Little, R./Rubin, D. (2019). Statistical Analysis with Missing Data, Third Edition. Wiley.

2 Histograms of IH Item Responses and Total Score

Supplementary Figure 1.

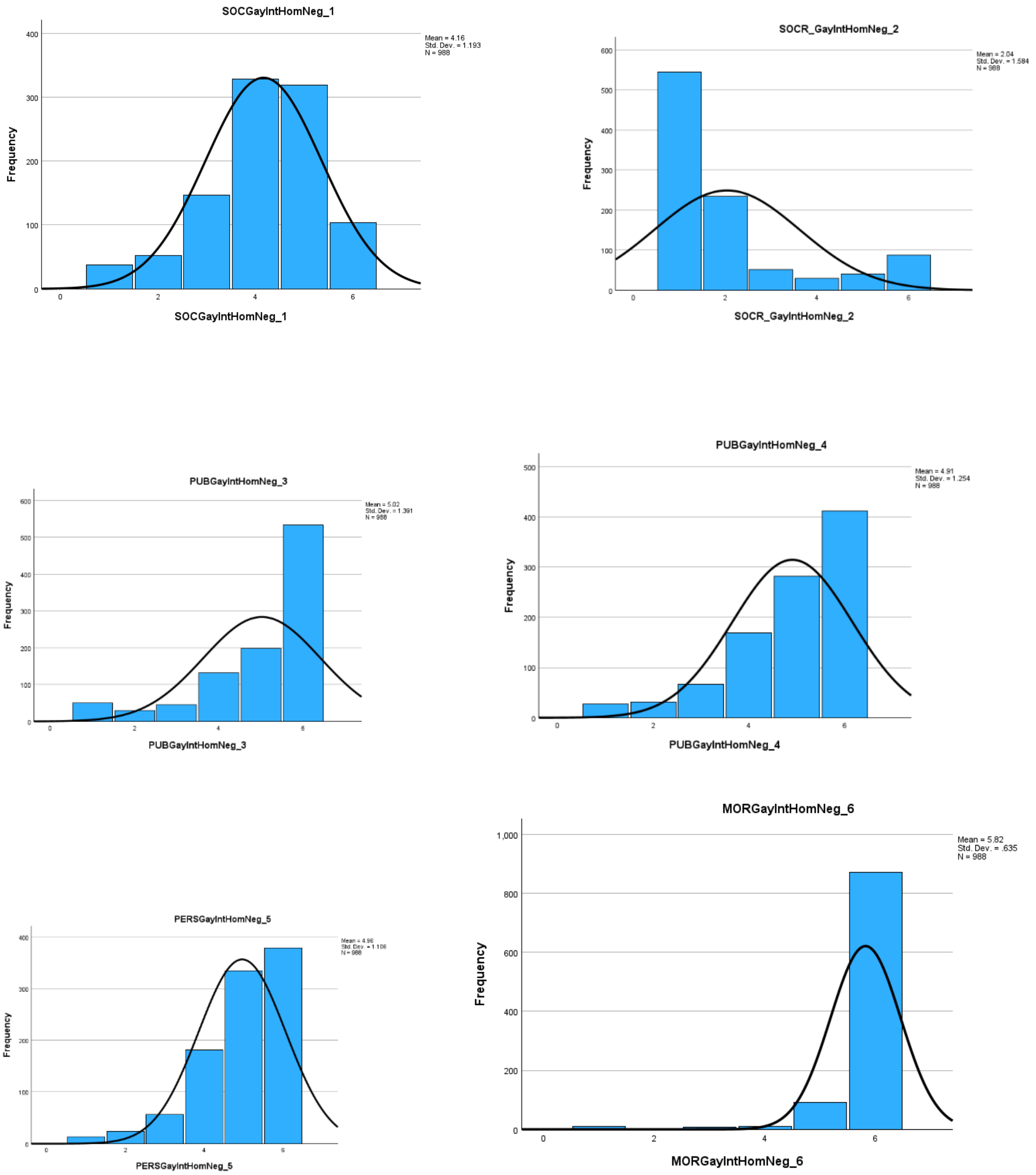

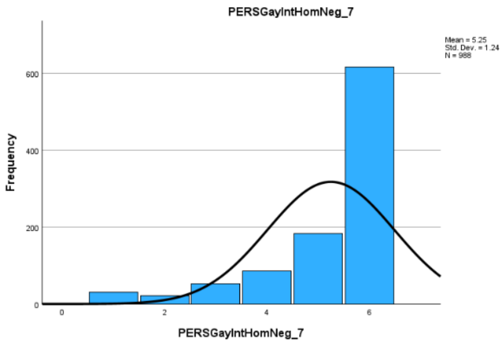
PERSGayIntHomNeg_7
Mean = 5.25
Std. Dev. = 1.24
N = 988
Frequency
600
400
200
0
0
2
4
6
PERSGayIntHomNeg_7

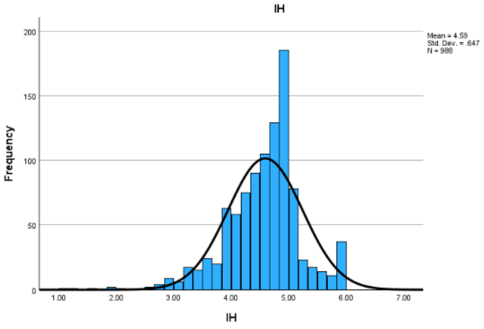
IH
Mean = 4.59
Std. Dev. = .647
N = 988
Frequency
200
150
100
50
0
1.00
2.00
3.00
4.00
5.00
6.00
7.00
IH
